# An International Expert Delphi Consensus on Defining Textbook Outcome in Liver Surgery (TOLS)

**DOI:** 10.1097/SLA.0000000000005668

**Published:** 2022-08-10

**Authors:** Burak Görgec, Andrea Benedetti Cacciaguerra, Timothy M. Pawlik, Luca A. Aldrighetti, Adnan A. Alseidi, Umberto Cillo, Norihiro Kokudo, David A. Geller, Go Wakabayashi, Horacio J. Asbun, Marc G. Besselink, Daniel Cherqui, Tan To Cheung, Pierre-Alain Clavien, Claudius Conrad, Mathieu D’Hondt, Ibrahim Dagher, Christos Dervenis, John Devar, Elijah Dixon, Bjørn Edwin, Mikhail Efanov, Giuseppe M. Ettore, Alessandro Ferrero, Constantino Fondevilla, David Fuks, Felice Giuliante, Ho-Seong Han, Goro Honda, Oscar Imventarza, David A. Kooby, Peter Lodge, Santiago Lopez-Ben, Marcel A. Machado, Hugo P. Marques, Nick O’Rourke, Juan Pekolj, Antonio D. Pinna, Nazario Portolani, John Primrose, Fernando Rotellar, Andrea Ruzzenente, Erik Schadde, Ajith K. Siriwardena, Sameer Smadi, Olivier Soubrane, Kenneth K. Tanabe, Catherine S.C. Teh, Guido Torzilli, Thomas M. Van Gulik, Marco Vivarelli, Stephen J. Wigmore, Mohammad Abu Hilal

**Affiliations:** * Department of Surgery, Poliambulanza Foundation Hospital, Brescia, Italy; † Department of Surgery, AmsterdamUMC, University of Amsterdam, Amsterdam, the Netherlands; ‡ Cancer Center Amsterdam, Amsterdam, the Netherlands; § Department of Surgery, University Hospital Southampton NHS Foundation Trust, Southampton, UK; ∥ Hepatobiliary and Abdominal Transplantation Surgery, Department of Experimental and Clinical Medicine, Riuniti Hospital, Polytechnic University of Marche, Ancona, Italy; ¶ Division of Surgical Oncology, Department of Surgery, The Ohio State University Wexner Medical Center and James Comprehensive Cancer Center, Columbus, OH; # Hepatobiliary Surgery Division, IRCCS San Raffaele Hospital, Milan, Italy; ** Department of Surgery, University of California San Francisco, San Francisco, CA; †† Department of Surgery, Oncology and Gastroenterology, Hepatobiliary Surgery and Liver Transplantation Unit, Padova University Hospital, Padova, Italy; ‡‡ Department of Surgery, Hepatobiliary Pancreatic Surgery Division, National Center for Global Health and Medicine, Tokyo, Japan; §§ Department of Surgery, University of Pittsburgh Medical Center, Pittsburgh, PA; ∥∥ Center for Advanced Treatment of Hepatobiliary and Pancreatic Diseases, Ageo Central General Hospital, Saitama, Japan; ¶¶ Hepato-Biliary and Pancreas Surgery, Miami Cancer Institute, Miami, FL; ## Department of Hepatobiliary Surgery, Paul Brousse University Hospital, Villejuif, France; *** Department of Surgery, The University of Hong Kong, Hong Kong, China; ††† Department of surgery and transplantation, University Hospital Zurich, Switzerland; ‡‡‡ Department of Surgery, St. Elizabeth’s Medical Center, Tufts University School of Medicine, Boston, MA; §§§ Department of Digestive and Hepatobiliary/Pancreatic Surgery, Groeninge Hospital, Kortrijk, Belgium; ∥∥∥ Department of Minimally Invasive Digestive Surgery, Antoine Béclère Hospital, Clamart, France; ¶¶¶ Department of Surgery, Konstantopouleio General Hospital, Nea Ionia, Athens, Greece; ### Department of Surgery, School of Clinical Medicine, Faculty of Health Sciences, University of the Witwatersrand, Johannesburg, South Africa; **** Hepato-Pancreatico-Biliary Unit, Department of General Surgery, Chris Hani Baragwanath Academic Hospital, Johannesburg, South Africa; †††† Department of Surgery, University of Calgary, Calgary, AB, Canada; ‡‡‡‡ Department of Hepato-Pancreato-Biliary Surgery and The Intervention Center, Oslo University Hospital Oslo, Oslo, Norway; §§§§ Institute of Clinical Medicine, Medical Faculty, University of Oslo, Oslo, Norway; ∥∥∥∥ Department of Hepato-Pancreato-Biliary Surgery, Moscow Clinical Research Centre, Moscow, Russia; ¶¶¶¶ General Surgery and Transplantation Unit, San Camillo Hospital, Rome, Italy; #### Department of General and Oncological Surgery, Umberto I Mauriziano Hospital, Turin, Italy; ***** General & Digestive Surgery, Hospital Universitario La Paz, IdiPAZ, Madrid, Spain; ††††† Department of Digestive, Oncologic and Metabolic Surgery, Institut Mutualiste Montsouris, Université Paris Descartes, Paris, France; ‡‡‡‡‡ Chirurgia Epatobiliare, Università Cattolica del Sacro Cuore-IRCCS, Rome, Italy; §§§§§ Department of Surgery, Seoul National University Bundang Hospital, Seoul National University College of Medicine, Seongnam-si, Gyeonggi-do, Republic of Korea; ∥∥∥∥∥ Department of Surgery, Institute of Gastroenterology, Tokyo Women’s Medical University, Tokyo, Japan; ¶¶¶¶¶ Department of surgery, Hospital Argerich, Buenos Aires, Argentina; ##### Department of surgery, Hospital Garrahan, Buenos Aires, Argentina; ****** Department of Surgery, Emory University School of Medicine, Winship Cancer Institute, Atlanta, GA; †††††† HPB and Transplant Unit, St James’s University Hospital, Leeds, UK; ‡‡‡‡‡‡ Department of General and Digestive Surgery, HPB Unit, Hospital Universitari de Girona Dr. Josep Trueta, Girona, Spain; §§§§§§ Department of Surgery, University of São Paulo, São Paulo, Brazil; ∥∥∥∥∥∥ Department of Surgery, Curry Cabral Hospital, Lisbon, Portugal; ¶¶¶¶¶¶ Department of HPB Surgery, Royal Brisbane and Women’s Hospital, Brisbane, Queensland, Australia; ###### Division of HPB Surgery and Liver Transplant Unit, Department of General Surgery, Hospital Italiano de Buenos Aires, Buenos Aires, Argentina; ******* Abdominal Transplant and HPB Center, Cleveland Clinic Florida, Weston, Florida; ††††††† Department of Clinical and Experimental Sciences, Surgical Clinic, University of Brescia, Italy; ‡‡‡‡‡‡‡ Department of General and Digestive Surgery, Clinica Universidad de Navarra, Pamplona, Spain; §§§§§§§ Department of Surgery, University of Verona, Verona, Italy; ∥∥∥∥∥∥∥ Institute of Physiology, University of Zurich, Zurich, Switzerland; ¶¶¶¶¶¶¶ Department of Surgery, Cantonal Hospital Winterthur, Zurich, Switzerland; ####### Division of Transplant Surgery, Department of Surgery, Rush University Medical Center, Chicago, IL; ******** Hepatobiliary and Pancreatic Surgery Unit, Manchester University NHS FT, Manchester, UK; †††††††† Department of Surgery, King Hussein Medical Center, Amman, Jordan; ‡‡‡‡‡‡‡‡ Department of Hepatobiliopancreatic Surgery, APHP, Beaujon Hospital, Clichy, France; §§§§§§§§ Division of Surgical Oncology, Department of Surgery, Massachusetts General Hospital, Harvard Medical School, Boston, MA; ∥∥∥∥∥∥∥∥ Section of Hepatobiliary Pancreatic Surgery, Surgical Oncology, and Minimally Invasive Surgery, St Luke’s Medical Center, Quezon City, Philippines; ¶¶¶¶¶¶¶¶ Division of Hepatobiliary and General Surgery, Department of Surgery, Humanitas Clinical and Research Center, IRCCS, Humanitas University, Rozzano, Italy; ######## Department of Hepato-Pancreato-Biliary (HPB)/Transplant Surgery, The University of Edinburgh Clinical Surgery, Edinburgh, UK

**Keywords:** composite measure, laparoscopic liver surgery, liver surgery, minimally invasive liver surgery, patient outcome, quality of care, robotic liver surgery, textbook outcome

## Abstract

To reach global expert consensus on the definition of TOLS in minimally invasive and open liver resection among renowned international expert liver surgeons using a modified Delphi method.

**Background:**

Textbook outcome is a novel composite measure combining the most desirable postoperative outcomes into one single measure and representing the ideal postoperative course. Despite a recently developed international definition of Textbook Outcome in Liver Surgery (TOLS), a standardized and expert consensus-based definition is lacking.

**Methods:**

This international, consensus-based, qualitative study used a Delphi process to achieve consensus on the definition of TOLS. The survey comprised 6 surgical domains with a total of 26 questions on individual surgical outcome variables. The process included 4 rounds of online questionnaires. Consensus was achieved when a threshold of at least 80% agreement was reached. The results from the Delphi rounds were used to establish an international definition of TOLS.

**Results:**

In total, 44 expert liver surgeons from 22 countries and all 3 major international hepato-pancreato-biliary associations completed round 1. Forty-two (96%), 41 (98%), and 41 (98%) of the experts participated in round 2, 3, and 4, respectively. The TOLS definition derived from the consensus process included the absence of intraoperative grade ≥2 incidents, postoperative bile leakage grade B/C, postoperative liver failure grade B/C, 90-day major postoperative complications, 90-day readmission due to surgery-related major complications, 90-day/in-hospital mortality, and the presence of R0 resection margin.

**Conclusions:**

This is the first study providing an international expert consensus-based definition of TOLS for minimally invasive and open liver resections by the use of a formal Delphi consensus approach. TOLS may be useful in assessing patient-level hospital performance and carrying out international comparisons between centers with different clinical practices to further improve patient outcomes.

There is an increasing demand for information about hospital quality of care, especially among patients undergoing complex surgical procedures.^1^ Conventional quality measurement has relied on assessing individual outcome variables such as morbidity, mortality, and hospital length of stay (LOS).^2^–^4^ Although these single outcome variables provide significant information and are useful for targeted quality improvement programs, they do not capture the multidimensional aspect of the surgical care pathway.^5^,^6^ Furthermore, small sample sizes and low event rates conspire to limit the precision of hospital outcome measures.^6^–^8^ In addition, it is difficult to use single outcome variables to compare the quality of care among hospitals, as any given institution may have a high score on 1 outcome, but low score on another. Therefore, composite measures have been suggested to be superior to individual outcome variables combining the multidimensional aspect of the complex surgical process into 1 single indicator.^9^–^15^

Textbook outcome (TO) is a novel composite measure firstly described in the field of gastrointestinal cancer surgery.^6^,^16^ It provides a comprehensive summary of hospital quality of care with special attention to patient-centered care.^17^ TO combines the most desirable postoperative outcomes into 1 single measure and embodies the “ideal” postoperative course. If a patient meets all the desirable postoperative outcomes, TO is achieved.^6^ In addition, TO represents a more holistic approach to quality assessment that may represent a better means to assess variation in performance and postoperative outcomes among various hospitals.^14^

To date, TO has been examined relative to several surgical specialities including liver surgery. Most definitions of TO in the field of liver surgery have been based on the opinions of a single expert or a small group of surgeons. Previously, our group proposed the first international definition of TO in Liver Surgery (TOLS) for laparoscopic and open liver resection (OLR) through an international single-round survey among all members of the European-African and International Hepato-Pancreato-Biliary Association and validated this definition in a large cohort.^18^ It is crucial, however, to refine and validate this proposed TOLS definition among a broader population of expert liver surgeons using an evidence-based consensus methodology. To that end, the aim of the current study was to define a global expert consensus on the definition of TOLS in minimally invasive liver resection (MILR) and OLR among renowned international expert liver surgeons using a modified Delphi method.

## METHODS

### Modified Delphi Process

The modified Delphi process in the current study took place between July 2020 and October 2021 and consisted of a 4-round web-based questionnaire in accordance with Conducting and Reporting Delphi Studies guidelines.^19^ The Delphi methodology aims to systematically survey a panel of experts to obtain consensus on specific questions or statements. This method has been widely and successfully used in several surgical specialties.^20^–^22^

### Expert Panel

Potential expert panel members were selected based on the possession of theoretical knowledge and extensive practical experience combined with significant scientific achievements in the field of MILR and/or OLR. Individuals were identified among surgeons from high-volume centers, representatives of Hepato-Pancreato-Biliary (HPB) societies, editorial boards of high-impact journals, and coauthors of high-impact publications. The expert panel was chosen to ensure contribution from the 3 HPB regions (ie, Europe/Africa/Middle East, Americas, and the Asian/Pacific region). A recruitment letter was sent via e-mail to all potential panelists. A total of 52 expert liver surgeons were invited and consented to participate in the Delphi process.

### Delphi Questionnaire

Three authors (B.G., A.B.C., and M.A.H.) designed the initial questionnaire and guided the Delphi process through all rounds. These individuals were responsible for collecting and organizing data, communicating with experts/committee members, and creating and distributing the electronic questionnaires. A steering committee consisting of 7 international expert liver surgeons (T.M.P., L.A.A., A.A.A., D.G., U.C., N.K., and G.W.), a well-distributed representation from all the 3 HPB continents, evaluated and approved each round of the questionnaire before dissemination to the expert panel. A literature review identifying all surgical outcome variables that could be included in the definition of TOLS was conducted. The initial survey comprised 6 surgical domains with a total of 19 questions on individual surgical outcome variables. A dichotomous (ie, yes/no or agree/disagree), multiple-choice, and open-ended polling method was chosen over a Likert scale, as the final goal was to assess whether there was agreement with the inclusion of certain surgical variables in the definition of TOLS or not. The binary system would force the experts to be more definitive in their responses. Within each survey domain, a section for comments was available, providing the opportunity to elaborate or explain responses. All responses were reviewed in an anonymous manner. Consensus on a question/statement was achieved when a threshold of at least 80% agreement was reached. In the fourth and last round, a moderate agreement rate of 60% to 80% was also considered a consensus. The online questionnaire was pilot tested by 2 surgeons who were not members of the expert panel. The questionnaires were emailed using Google Forms Survey (Google; Mountain View, CA) over 4 rounds.

### Delphi Rounds

The first round questionnaire collected information and opinions on surgeon demographics, surgical experience, current knowledge of composite measures, and current application of TO. A short explanation video on the concept of TO (Supplemental Video 1, Supplemental Digital Content 1, http://links.lww.com/SLA/E167) was developed and included in the first Delphi round to ensure that all panelists were familiar with the concept of TO. Questions that reached consensus in the first round were not sent for a second round. Questions with <80% agreement were returned to the Steering Committee to be evaluated. The Steering Committee had the option to revise or discard questions based on feedback from the panelists. The agreement rates per question and statement obtained in the first round together with the expert’s comments were incorporated into a second round. In round 2, the experts were offered the opportunity to view the group results from the first round and change their own response. In the third and fourth round, questions in the surgical domains without agreement in previous rounds were separated and re-presented to the experts. The final definition of TOLS included all individual surgical variables that reached consensus during the entire Delphi process.

### Statistical Analysis

Data analyses was based on percentage response rates for each statement or question in each round of the Delphi process. Data were visualized in Google Forms Survey. An online TOLS calculator was developed and made available on http://www.evidencio.com, an online platform for medical decision models.

## RESULTS

Among the 52 experts invited, 44 (84.6%) completed round 1. Of these 44, 42 completed round 2 (95.5%). Of these 42, 41 completed round 3 and 4 (97.6%). Table 1 presents the demographic characteristics of all expert panel members. Two third of all participants resided in Europe/Africa/Middle East (n=28; 63.6%). The majority of panelists were at the rank of professor (n=33, 75%). Forty experts (90.9%) had experience in both MILR and OLR, whereas 4 experts (9.1%) indicated no experience performing MILR. The median annual hospital volume of MILR was 60 resections [interquartile range (IQR): 30–88 resections], whereas the median annual hospital volume of OLR was 100 resections (IQR: 50–154 resections). The median annual volume of MILR per surgeon was 30 resections (IQR: 10–54 resections). The median annual volume of OLR per surgeon was 40 resections (IQR: 20–68 resections).

**TABLE 1:** Characteristics of Expert Panel Members

Characteristics	Expert Panel Members N=44
Sex, n (%)	
Male	43 (97.8)
Female	1 (2.2)
Distribution in HPB society continents, n (%)	
Europe/Africa/Middle East	28 (63.6)
Americas	10 (22.7)
Asian Pacific	6 (13.6)
Country of residency, n (%)	
Argentina	2 (4.5)
Australia	1 (2.3)
Belgium	1 (2.3)
Brazil	1 (2.3)
Canada	1 (2.3)
France	4 (9.1)
Greece	1 (2.3)
Hong Kong	1 (2.3)
Italy	8 (18.2)
Japan	1 (2.3)
Jordan	1 (2.3)
Norway	1 (2.3)
Philippines	1 (2.3)
Portugal	1 (2.3)
Russia	1 (2.3)
South Africa	1 (2.3)
South Korea	1 (2.3)
Spain	3 (6.8)
Switzerland	1 (2.3)
The Netherlands	2 (4.5)
United Kingdom	3 (6.8)
United States	6 (13.6)
Current highest degree	
Professor	33 (75)
PhD degree	9 (20.5)
Medical degree	2 (4.5)
Employment at type of medical center, n (%)	
University	27 (61.4)
University affiliated	3 (6.8)
Community	14 (31.8)
Individual experience with MILS, n (%)	40 (90.9)
Annual hospital volume of MILR, median (IQR)	60 (30–88)
Annual hospital volume of open liver surgery, median (IQR)	100 (50–154)
Annual individual volume of MILR, median (IQR)	30 (10–54)
Annual individual volume of open liver surgery, median (IQR)	40 (20–68)

Values in parentheses are percentages unless mentioned otherwise. Percentages may not add up due to rounding and missing data.

### Surgical Quality Assessment

Most surgeons (n=32; 72.2%) indicated that they were currently using multiple individual surgical outcome variables such as morbidity, mortality, and LOS to assess the quality of surgical care in their center. Two surgeons were using benchmarking (4.5%), whereas 1 surgeon (2.3%) was using TO measure the quality of liver surgical care in their center. Experts agreed that composite measures better reflect the multidimensional aspect of the surgical process more than an individual outcome variable (agreement rate 81.8%) and should be used to assess quality of care in liver surgery (agreement rate 81.1%). TO was deemed a useful composite measure to assess quality relative to liver surgery in a single center (agreement rate 92.2%), as well as to compare postoperative outcomes of liver surgery between hospitals (agreement rate 90.5%). There was good agreement that TO is a useful tool for determining which surgical outcome variable is the most limiting factor in achieving the ideal postoperative course and initiating targeted quality improvement programs (agreement rate 90.5%). Furthermore, experts agreed that TO will be instrumental in improving quality of liver surgery on a national (agreement rate 90.5%), as well as international level (agreement rate 88.1%). As no consensus was reached on the need to define TOLS for MILR and OLR separately (agreement rate 78.6%), an overall definition of TOLS for MILR and OLR was developed. Supplemental Table 1, Supplemental Digital Content 2, http://links.lww.com/SLA/E168 shows the agreement rates per statement in rounds 1 and 2.

### Definition of TOLS

**FIGURE 1:**
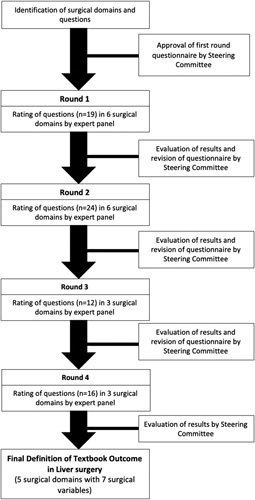
Flowchart of the Delphi process.

Supplemental Table 2, Supplemental Digital Content 2, http://links.lww.com/SLA/E168 shows a summary of the 4-round Delphi process with questions per domain that were essential to arrive at the final definition of TOLS (Fig. 1). Of note, the number of questions where consensus was achieved improved for each domain from rounds 1 to 4.

In rounds 1 and 2, there was consensus for questions in the domains: Intraoperative Incidents, Mortality and Oncological Resection Margin. Questions in the domain General Postoperative Complications, Liver Surgery-related Complications and Length of Hospital Stay did not reach consensus after rounds 1 and 2 and were revised and submitted for voting in round 3. Within all domains, the surgical variables unplanned intensive care admission (agreement rate 21.4%), postoperative (surgical/endoscopic/radiologic) reintervention (agreement rate 59.5%), postoperative ascites solely (agreement rate 20.5%), and R1 vascular resection (agreement rate 64.3%) had a low agreement rate after rounds 1 and 2 and were excluded from any further voting in round 3.

In round 3, experts approved that the surgical variables postoperative complications and readmission in the domain General Postoperative Complications should be included in the definition of TOLS, yet no consensus was achieved on the time frame and grading. Furthermore, in round 3, based on expert’s comments, LOS was redefined as time to functional recovery, excluding any prolonged LOS due to cultural or social reasons. There was no consensus and a high variation in the proportion of answers for the maximal acceptable LOS in MILR and OLR stratified for minor and major resections. Therefore, the Steering Committee decided to exclude questions surveying the maximally LOS from round 4 and propose the additional development of an extended definition of TOLS including prolonged LOS (TOLS+).

In round 4, questions in the unagreed domains were revised and separated. Eventually, there was agreement in all surgical domains.

Overall, the expert panel achieved consensus on the inclusion of 5 surgical domains in the main definition of TOLS (Table 2). TOLS was defined as the absence of intraoperative grade ≥2 incidents (defined according to the Oslo classification),^23^ postoperative bile leak of grade B or C (according to the severity grading of the International Study Group of Liver Surgery),^24^ postoperative liver failure grade B or C (according to the severity grading of the International Study Group of Liver Surgery),^25^ major postoperative complications within 90 days (Clavien–Dindo grade III or higher),^26^ readmission within 90 days after discharge due to surgery-related major complications (Clavien–Dindo Grade III or higher), in-hospital or 90-day mortality, and the presence of R0 resection margin (ie, 1mm or more tumor-free margin). An online calculator for TOLS is available via https://www.evidencio.com/models/show/2794. Furthermore, the expert panel agreed on the development of an extended definition of TOLS including prolonged LOS (TOLS+). TOLS+ includes the same variables as TOLS but adds “prolonged LOS.” On the basis of the survey results, overall prolonged LOS was defined as >3 days for minor MILR, >5 days for major MILR, >5 days for minor OLR, and >10 days for major OLR. A subdivision of TOLS+ per HPB society region showed that for Europe/Africa/Middle East, prolonged LOS was defined as >3 days for minor MILR, >7 days for major MILR, >5 days for minor OLR, and >10 days for major OLR. For Americas, prolonged LOS was defined as >3 days for minor MILR, >5 days for major MILR, >5 days for minor OLR, and >8 days for major OLR. For Asian Pacific, prolonged LOS included >5 days for minor MILR, >7 days for major MILR, >6 days for minor OLR, and >10 days for major OLR. The questions and respective agreement rates in each round of the Delphi processes are depicted in Supplemental Tables 3–5, Supplemental Digital Content 2, http://links.lww.com/SLA/E168.

**TABLE 2:** The Main Definition of Textbook Outcome in Liver Surgery (TOLS) Obtained Through the Delphi Process

Definition of Textbook Outcome in Liver Surgery	
Domain: intraoperative incidents	The absence of intraoperative incidents of grades 2 and 3 only
Domain: general postoperative complications	The absence of 90-day postoperative complications Clavien–Dindo III or higher
	The absence of 90-day readmission due to surgery-related complications Clavien–dindo Grade 3 or higher
Domain: liver surgery-related postoperative complications	The absence of postoperative bile leakage of grades B and C
	The absence of Postoperative liver failure of grades B and C
Domain: mortality	The absence of in-hospital and 90-day mortality
Domain: oncological resection margin	The absence of R1 and R2 resection margin for all malignant indications

## DISCUSSION

To the best of our knowledge, this is the first study undertaken as a cohesive effort to provide an international expert consensus-based definition of TOLS. A panel of 44 expert liver surgeons assessed a total of 26 questions in 6 surgical domains using a modified 4-round Delphi process and defined TOLS as the absence of intraoperative grade ≥2 incidents, postoperative bile leak of grade B/C, postoperative liver failure grade B/C, 90-day major postoperative complications, 90-day readmission due to surgery-related major complications, 90-day/in-hospital mortality, and the presence of R0 resection margin. In addition, the Delphi process developed the concept TOLS+, an extended definition of TOLS including prolonged LOS.

Previous studies focusing on the development of a definition of TO in the field of liver surgery have been scarce. Recently, our group obtained the first international survey-based definition of TOLS consisting of 6 surgical variables including the absence of intraoperative grade ≥2 incidents, postoperative bile leakage of grade B or C, major complications, readmission within 30 days after discharge, in-hospital mortality, and the presence of R0 resection margin.^18^ Of note, TOLS was defined and validated for laparoscopic liver resection and OLR only and other minimally invasive techniques such as robotic liver resections were not within the scope of this study. Furthermore, our previous study included a single-round survey and was disseminated to all liver surgeons worldwide without considering liver surgical experience. Another recently published study investigated trends in TO over time after complex gastrointestinal surgery for malignancies by assessing TO in a cohort of 94,324 patients.^27^ A former established definition of TO in the field of hepatopancreatic surgery including no perioperative complication, no prolonged LOS (> 75th percentile), no 90-day readmission, and no 90-day mortality was used.^14^ Of note, although this definition seems to be widely accepted, it is not reported how it was created. It might be based on the opinion of a small group of experts, increasing the possibility of individual bias. In addition, unlike the current study, this definition of TO did not include liver-specific complications and was not stratified for surgical approach.

One of the interesting findings of the current study is that all individual surgical variables in the definition of TOLS (ie, complications, readmission, mortality) were defined relative to 90 days as the most appropriate period in which an event should be evaluated. This is in line with previous studies that investigated the validity of 90-day outcomes compared with 30-day outcomes.^28^–^32^ A nationwide multicenter retrospective study examined the 30- and 90-day mortality of 2597 patients with colorectal liver metastases or hepatocellular carcinoma undergoing liver resection between 1991 and 2006 by assessing the incremental increase in mortality noted at 90 days and concluded that 30-day mortality does not completely reflect the postoperative mortality risk as compared with 90-day mortality.^32^ They demonstrated that calculating mortality based solely on data available at 30 days is deceptive, underestimating true perioperative mortality by up to 50%. Another study investigated outcomes in 969 patients undergoing radical cystectomy between 2011 and 2018 and found that 90-day complications were significantly higher as compared with 30-day complications.^29^ They concluded that assessing complications just at 30 days would miss a high number of major complications and deaths.

Although the Delphi process identified LOS as an important surgical variable to be included in TOLS, no consensus could be reached on the maximal LOS stratified for type of resection and surgical approach. Therefore, the main TOLS definition in the current study did not include prolonged LOS. This approach is in accordance with our previous study on TOLS, but in contrast with other TO definitions in the field of liver and other complex surgery.^6^,^16^,^18^,^33^–^35^ The current TOLS definition is an international definition; importantly, LOS is not only associated with functional recovery but also depends on differences in cultural interpretation and the organization of health care systems among countries. Recently, Merath et al^17^ assessed TO among patients undergoing curative-intent resection of intrahepatic cholangiocarcinoma and showed that the incidence of prolonged LOS was remarkably different among Eastern hospitals (74.3%) and western hospitals (33.3%). The current study proposed TOLS+ to be used on national level with predefined thresholds based on international opinion. However, it may be beneficial for centers in the same country or within the same health care system to reformulate thresholds for LOS in TOLS+ to be able to compare patient-level hospital performance on a nationwide scale.

The expert panel agreed on including radical resection (R0 resection margin) for all malignant indications as an oncological requisite for achieving TOLS. Previous studies confirmed that short-term oncological outcomes, such as resection margin, may be associated with recurrence-free and overall survival.^36^–^38^ It is worth noting, however, that in certain malignancies, R1 resection is inevitable and should not be seen as a surgical error, especially when R1 vascular resection is involved.^39^–^41^ The expert panel, on the other hand, did not approve R1 vascular resection to be included in the definition of TOLS. Radical resection remains the gold standard in the surgical treatment of liver malignancies. Furthermore, although the inclusion of oncological resection margin in TOLS may imply that it is only applicable for malignant indications, we would like to highlight that the international TOLS definition obtained in this Delphi process covers all indications and may be used for benign indications as well. The current study proposes TOLS for benign liver diseases, which includes the same variables as TOLS without oncological resection margin.

TOLS has several potential advantages as compared with the assessment of individual outcome variables and may be useful for many stakeholders. Marshall *et al* 42 demonstrated that patients rarely searched for information on hospital performance and, if sought, did not understand or trust it. Therefore, for patients, TOLS shows their odds of achieving the best outcome in a certain hospital presented as a summary measure. For surgeons, TOLS provides information on how often a certain liver surgical procedure is successful, which may enhance quality improvement. On a hospital level, it may be useful in overall interhospital comparisons as TOLS summarizes indicators on patient safety, effectiveness, and efficiency. For example, the annual TOLS rate could be calculated per center with the identification of the most limiting variable in achieving TOLS. Subsequently, an interhospital comparison of annual TOLS rates and the most limiting variables in achieving TOLS could be performed to identify differences. Centers could share their experiences and learn from each other how to improve a certain individual outcome variable within the TOLS definition, which may have a high rate in 1 center, but a low rate in another center, to eventually improve the overall TOLS rate in a certain center. Furthermore, by combining desirable individual outcomes in 1 comprehensive measure, TOLS precludes defensive, single indicator–driven practice. For example, a hospital policy to accept a certain readmission rate by discharging patients early to get a better score at the variable length of hospital stay may not be in the patients’ best interest.

Despite the remarkable technological developments in recent decades, the implementation of digital applications and artificial intelligence in the field of surgery is still limited as surgery consists of procedural multimodal data in a dynamic environment.^43^,^44^ Nevertheless, considering the increasing amount of surgical definitions and models, software applications and artificial intelligence are more than ever needed to ease and widen the application of TOLS. The development of an online calculator to score the 7 surgical outcome variables by the surgeon and determine whether a patient achieves TOLS or not might be a valuable first step in this process (https://www.evidencio.com/models/show/2794). Furthermore, we propose the use of machine learning and natural language processing to create integrated autonomous action within the field of TOLS. Individual outcome variables within the TOLS definition could be identified in the electronic patient record 90 days postoperatively using natural language processing. Subsequently, machine learning may aid in calculating whether a patient meets all requirements for TOLS and this could be translated in an overall TOLS rate. Future studies should focus on these principles.

The current study has several limitations. First, the panel comprised mainly experts from Europe/Africa/Middle East, whereas significant number of experts per HPB society continent were invited to participate. Nevertheless, a large number of panelists consisting of 44 expert liver surgeons with international experience and broad surgical view participated and maintained the generalizability of these results. Second, the expert panel shows a lack of sex diversity with only 1 female expert included. However, experts were selected based on their expertise without specifically focusing on sex. Third, our panel consisted of surgeons only and selection may have been skewed toward those with interest in composite measures such as TO. The current Delphi process lacked potentially important perspectives of clinicians from other disciplines (eg, interventional radiologists, hepatologists, anesthetists, and oncologists) involved in the multidisciplinary treatment of patients with malignant and benign liver diseases. The current study may encourage and inspire other disciplines to evaluate their outcome through composite measures such as TOLS. Fourth, the individual experience with MILR and the annual hospital volume of MILR and OLR are self-reported and may be overestimated. Therefore, these numbers should be interpreted carefully. Fifth, although the web-based Delphi consensus technique was the appropriate tool for bringing together views of experts on this topic, a virtual meeting would have been helpful to discuss questions that did not reach consensus after the third round and add nuance to agreed surgical domains. We did attempt to organize a virtual meeting, but less than half of the panelist from all around to world were able to attend the meeting because of differences in time zones and busy schedules related to the coronavirus disease 19 pandemic, limiting the possibility to reach consensus. Therefore, the virtual meeting was canceled. Sixth, TO lacks weighing of the different outcome variables included. However, TO is a composite measure with an “all or none” approach and this simplicity forms the base of TO. O’Brien *et al* 45 investigated 4 methods for combining indicators in adult cardiac surgery including an opportunity-based approach, weighted averaging of item-specific estimates, “all or none” scoring, and latent trait analysis, and showed that the “all or none” approach was the strongest for establishing a composite measure. Nevertheless, weighing of surgical variables might improve the concept of TO. Future studies should focus on this weighing as, currently, no clear data or literature from which to derive these weights is available.

## CONCLUSIONS

To the best of our knowledge, the current study presents the first international expert consensus-based definition of TOLS for MILR and OLR by the use of a formal consensus approach. TOLS may be useful in assessing patient-level hospital performance and carrying out international comparisons between centers with different clinical practices to aid the further improvement of outcomes for patients. Future large studies are warranted to validate this standardized and expert consensus-based TOLS definition to eventually support its widespread use in daily clinical practice.

## Supplementary Material
