# Supplementary material for: An International Expert Delphi Consensus on Defining Textbook Outcome in Liver Surgery (TOLS)
Source: Ann Surg. 2022 Aug 10;277(5):821–8. doi: 10.1097/SLA.0000000000005668 (PMC10082050; doi:10.1097/SLA.0000000000005668)
Supplement: Supplementary file 2 [file sla-277-00821-s002.docx]

**SUPPLEMENTAL MATERIAL**

**Supplemental Video 1.** An explanation video on the concept of Textbook Outcome included in round 1 of the Delphi process.

**Supplemental Table 1.** Statements with regards to composite measures and textbook outcome presented in round 1 and 2 with the corresponding agreement rates.

| **Statement** | **Agreement rate**  **Round 1** | **Agreement rate**  **Round 2** |
| --- | --- | --- |
| Composite measures reflect the multidimensional aspect of the surgical process more than an individual outcome indicator (i.e. morbidity and mortality). | 81.1% | - |
| We should use composite measures to assess the quality of our surgical care in liver surgery | 81.1% | - |
| Textbook outcome is a useful composite measure to asses quality in liver surgery in a single center | 75% | 92.9% |
| Textbook outcome is an useful tool to determine which surgical outcome parameter is the most limiting factor in achieving the ideal postoperative course and to initiate targeted quality improvement programs | 67% | 90.5% |
| Textbook outcome is a useful composite measure to compare postoperative outcomes of liver surgery between hospitals. | 73 % | 90.5% |
| Textbook outcome will be instrumental in improving quality of liver surgery on a nationwide scale | 75% | 90.5% |
| Textbook outcome will be instrumental in improving quality of liver surgery on an international level | 77% | 88.1% |
| Textbook outcome in liver surgery should be defined for open and minimally invasive surgery separately | 63% | 78.6% |

**Supplemental Table 2.** Summary of the 4-Round Delphi Consensus Process to arrive at the final definition of Textbook Outcome in Liver surgery (TOLS).

| **Question** | **Answer option/Variable descriptive with agreement rate in the subsequent rounds** | **Round 1** | **Round 2** | **Round 3** | **Round 4** |
| --- | --- | --- | --- | --- | --- |
| **Domain 1: Intraoperative incidents** | | | | | |
| Which intraoperative incident parameter should be included in the definition of Textbook Outcome in liver surgery? | The absence of intraoperative incidents of grade 2 & 3 only | 63.6% | 83.3%* |  |  |
| **Domain 2: Mortality** | | | | | |
| Which mortality parameter should be included in the definition of Textbook Outcome in liver surgery? | The absence of In-hospital and no 90-day mortality | 56.8% | 83.3%* |  |  |
| **Domain 3: General postoperative complications** | | | | | |
| ***Main definition*** | | | | | |
| Which general postoperative complication parameter should be included in the definition of Textbook Outcome in liver surgery? | The absence of Complication of Clavien-Dindo II or higher | 40.9% | 59.5% |  |  |
| Do you agree postoperative complications to be included in the definition of Textbook Outcome in liver surgery? | Yes | - | - | 100%* |  |
| Which Clavien-Dindo Grade or higher should be included in the definition of Textbook Outcome in liver surgery? | The absence of Complication of Clavien-Dindo III or higher | - | - | 63.4% |  |
| Should we consider Clavien-Dindo Grade 3 or higher with regards to postoperative complications? | Yes | - | - | - | 80.5%* |
| ***Period of postoperative complication*** |  |  |  |  |  |
| Should we consider 30-or 90-day postoperative complications in the definition of Textbook Outcome in liver surgery? | 90-day | - | - | 73.2% |  |
| Should we consider 90-day postoperative complications in the definition of Textbook Outcome in liver surgery? | Yes | - | - | - | 73.2%** |
| ***Readmission*** |  |  |  |  |  |
| Which other general postoperative complication parameter should be included in the definition of Textbook Outcome in open liver surgery | The absence of readmission within 90 days after discharge | 18.2% | 38.1% |  |  |
| Do you think Readmission should be included in the definition of Textbook Outcome in Liver Surgery? | Yes | - | - | 100%* |  |
| Should we consider Readmission due to all reasons or just surgery related complications? | Just surgery related complications of Clavien-Dindo III or higher | - | - | 31.7% |  |
| Within what period should we consider readmission as an event? | 90 days | - | - | 61% |  |
| Should we consider Readmission due to surgery related complications Clavien-dindo Grade 3 or higher? | Yes | - | - | - | 68.3%** |
| Should we consider Readmission within 90 days? | Yes | - | - | - | 63.4%** |
| **Domain 4: Liver surgery-related postoperative complications** |  |  |  |  |  |
| ***Postoperative bile leakage*** |  |  |  |  |  |
| Which postoperative bile leakage parameter should be included in the definition of Textbook Outcome in liver surgery? | The absence of Postoperative bile leakage of grade B & C | 59.1% | 76.2% |  |  |
| Which postoperative bile leakage grading should be included to reach Textbook Outcome in Liver Surgery. | The absence of bile leakage of grade B & C | - | - | 61% |  |
| Should we consider bile leakage grade B and C? | Yes | - | - | - | 87.8%** |
| ***Postoperative liver failure*** |  |  |  |  |  |
| Which postoperative liver failure grading parameter should be included in the definition of Textbook Outcome in open liver surgery? | The absence of Postoperative liver failure of grade B & C | - | 66.7% |  |  |
| Which grading of postoperative liver failure should be included to reach Textbook Outcome in Liver Surgery? | The absence of Postoperative liver failure of grade B & C | - | - | 70.7% |  |
| Should we consider liver failure grade B and C? | Yes |  |  |  | 90.2%** |
| **Domain 5: Length of Hospital Stay** |  |  |  |  |  |
| Should length of hospital stay be a parameter for achieving Textbook Outcome in liver surgery? | Yes | 77.3% | 83.3%* |  |  |
| Should length of hospital stay separated for minor and major liver resections? | Yes | 81.1% | 92.9%* |  |  |
| What should be the maximally length of stay for achieving Textbook Outcome after minimally invasive minor liver resection? | Less than or equal to 3 days | - | 28.6% | 56.1% | - |
| What should be the maximally length of stay for achieving Textbook Outcome after minimally invasive major liver resection? | Less than or equal to 5 days |  | 16.7% | 73.2% |  |
| What should be the maximally length of stay for achieving Textbook Outcome after open minor liver resection? | less than or equal to 5 days |  | 40.5% | 46.3% |  |
| What should be the maximally length of stay for achieving Textbook Outcome after open major liver resection? | less than or equal to 10 days |  | 46.3% | 51.2% |  |
| Length of stay should not be included in the main definition of TOLS. | Agree | - | - | - | 73.2%** |
| Length of stay should be included in an extended definition of TOLS (TOLS+). | Agree | - | - | - | 70.7%** |
| **Domain 6: Oncological resection Margin** |  |  |  |  |  |
| Which resection margin parameter should be included in the definition of Textbook Outcome in MILS? | The absence of R1 Resection AND R2 Resection | 59.1% | 92.9%* |  |  |
| Should oncological resection margin be included for all or selected malignant indications (multiple selections possible)? | All malignant indications | 88.6% | 90.5%* |  |  |

* Consensus achieved in round 1-3 (i.e. >80% of the experts agree)

** Consensus achieved in round 4(i.e. >60% of the experts agree)

**Supplemental Table 3.** Domains and questions presented in round 1 and 2 with the corresponding agreement rates.

| **Question** | **Answer option** | **Agreement rate**  **Round 1** | **Agreement rate**  **Round 2** |
| --- | --- | --- | --- |
| **Domain 1: Intraoperative incidents** |  |  |  |
| Q1. Which intraoperative incident parameter should be included in the definition of Textbook Outcome in *minimally invasive* liver surgery? | - The absence of intraoperative incidents of all grades | 27.3% | 9.5% |
|  | - The absence of intraoperative incidents of grade 2 & 3 only | 63.6% | 83.3% |
|  | - The absence of intraoperative incidents of grade 3 only | 6.8% | 2.4% |
|  | - Intraoperative incidents should not be included in Textbook Outcome | 2.3% | 4.8% |
| Q2. Which intraoperative incident parameter should be included in the definition of Textbook Outcome in *open* liver surgery? | - The absence of intraoperative incidents of all grades | 25% | 9.5% |
|  | - The absence of intraoperative incidents of grade 2 & 3 only | 63.3% | 83.3% |
|  | - The absence of intraoperative incidents of grade 3 only | 6.8% | 2.4% |
|  | - Intraoperative incidents should not be included in Textbook Outcome | 4.5% | 4.8% |
| **Domain 2: Mortality** |  |  |  |
| Q3. Which mortality parameter should be included in the definition of Textbook Outcome in *minimally invasive* liver surgery? | - The absence of in-hospital mortality | 2.3% | 2.4% |
|  | - The absence of 30-day mortality | 2.3% | 2.4% |
|  | - The absence of In-hospital and no 30-day mortality | 13.6% | 4.8% |
|  | - The absence of 90-day mortality | 22.7% | 7.1% |
|  | - The absence of In-hospital and no 90-day mortality | 56.8% | 83.3% |
|  | - Mortality should not be included in Textbook Outcome | 2.3% | 0% |
| Q4. Which mortality parameter should be included in the definition of Textbook Outcome in *open liver* surgery? | - The absence of in-hospital mortality | 2.3% | 2.4% |
|  | - The absence of 30-day mortality | 2.3% | 2.4% |
|  | - The absence of In-hospital and no 30-day mortality | 13.6% | 2.4% |
|  | - The absence of 90-day mortality | 22.7% | 7.1% |
|  | - The absence of In-hospital and no 90-day mortality | 56.8% | 85.7% |
|  | - Mortality should not be included in Textbook Outcome | 2.3% | 0 |
| **Domain 3: General postoperative complications** |  |  |  |
| Q5. Which general postoperative complication parameter should be included in the definition of Textbook Outcome in MILS? | - The absence of Complication of Clavien-Dindo IV or higher | 0% | 2.4% |
|  | - The absence of Complication of Clavien-Dindo III or higher | 31.8% | 31% |
|  | - The absence of Complication of Clavien-Dindo IIIa or higher | 13.6% | 7.1% |
|  | - The absence of Complication of Clavien-Dindo IIIb or higher | 6.8% | 0% |
|  | - The absence of Complication of Clavien-Dindo II or higher | 40.9% | 59.5% |
|  | - Postoperative complication should not be included in Textbook Outcome | 6.8% | 0% |
| Q6. Which general postoperative complication parameter should be included in the definition of Textbook Outcome in open liver surgery? | - The absence of Complication of Clavien-Dindo IV or higher | 0% | 2.4% |
|  | - The absence of Complication of Clavien-Dindo III or higher | 31.8% | 31% |
|  | - The absence of Complication of Clavien-Dindo IIIa or higher | 13.6% | 7.1% |
|  | - The absence of Complication of Clavien-Dindo IIIb or higher | 6.8% | 0% |
|  | - The absence of Complication of Clavien-Dindo II or higher | 40.9% | 59.5% |
|  | - Postoperative complication should not be included in Textbook Outcome | 6.8% | 0% |
| Q7. Which other general postoperative complication parameter should be included in the definition of Textbook Outcome in MILS? | - The absence of unplanned intensive care admission | 6.8% | 21.4% |
|  | - The absence of reintervention (surgical / endoscopic / radiological) | 38.6% | 59.5% |
|  | - The absence of readmission within 30 days after discharge | 18.2% | 33.3% |
|  | - The absence of readmission within 90 days after discharge | 18.2% | 38.1% |
|  | - None of the above | 18.2% | 7.1% |
| Q8. Which other general postoperative complication parameter should be included in the definition of Textbook Outcome in open liver surgery? | - The absence of unplanned intensive care admission | 6.8% | 21.4% |
|  | - The absence of reintervention (surgical / endoscopic / radiological) | 38.6% | 64.3% |
|  | - The absence of readmission within 30 days after discharge | 18.2% | 28.6% |
|  | - The absence of readmission within 90 days after discharge | 18.2% | 38.1% |
|  | - None of the above | 18.2% | 7.1% |
| **Domain 4: Liver surgery-related postoperative complications** |  |  |  |
| Q9. Which postoperative bile leakage parameter should be included in the definition of Textbook Outcome in MILS? | - The absence of Postoperative bile leakage of all grades | 29.5% | 23.8% |
|  | - The absence of Postoperative bile leakage of grade B & C | 59.1% | 76.2% |
|  | - The absence of Postoperative bile leakage of grade C | 6.8% | 0% |
|  | - Postoperative bile leakage should not be included in Textbook Outcome | 4.5% | 0% |
| Q10. Which postoperative bile leakage parameter should be included in the definition of Textbook Outcome in open liver surgery? | - The absence of Postoperative bile leakage of all grades | 29.5% | 23.8% |
|  | - The absence of Postoperative bile leakage of grade B & C | 59.1% | 76.2% |
|  | - The absence of Postoperative bile leakage of grade C | 6.8% | 0% |
|  | - Postoperative bile leakage should not be included in Textbook Outcome | 4.5% | 0% |
| Q11. (Round 1) Which other postoperative liver surgery-related complication should be included in the definition of Textbook Outcome in MILS? | - The absence of postoperative liver failure | 68.2% | - |
|  | - The absence of postoperative ascites | 20.5% | - |
|  | - None of the above | 11.4% | - |
| Q12. (Round 1) Which other postoperative liver surgery-related complication should be included in the definition of Textbook Outcome in open liver surgery? | - The absence of postoperative liver failure | 68.2% | - |
|  | - The absence of postoperative ascites | 20.5% | - |
|  | - None of the above | 11.4% | - |
| Q13. (Round 2) Which postoperative liver failure grading parameter should be included in the definition of Textbook Outcome in MILS? | - The absence of Postoperative liver failure of all grades | - | 23.8% |
|  | - The absence of Postoperative liver failure of grade B & C | - | 66.7% |
|  | - The absence of Postoperative liver failure of grade C | - | 7.1% |
|  | - Postoperative liver failure should not be included in Textbook Outcome | - | 2.4% |
| Q14. (Round 2) Which postoperative liver failure grading parameter should be included in the definition of Textbook Outcome in open liver surgery? | - The absence of Postoperative liver failure of all grades |  | 23.8% |
|  | - The absence of Postoperative liver failure of grade B & C |  | 66.7% |
|  | - The absence of Postoperative liver failure of grade C |  | 7.1% |
|  | - Postoperative liver failure should not be included in Textbook Outcome |  | 2.4% |
| **Domain 5: Length of Hospital Stay** |  |  |  |
| Q15. Should length of hospital stay be a parameter for achieving Textbook Outcome in MILS? | - Yes | 77.3% | 83.3% |
|  | - No | 22.7% | 16.7% |
| Q16. Should length of hospital stay be a parameter for achieving Textbook Outcome in open liver surgery? | - Yes | 70.5% | 83.3% |
|  | - No | 29.5% | 16.7% |
| Q17. Should length of hospital stay separated for minor and major liver resections in MILS? | - Yes | 81.1% | 92.9% |
|  | - No | 18.9% | 7.1% |
| Q18. Should length of hospital stay separated for minor and major liver resections in MILS? | - Yes | 81.1% | 92.9% |
|  | - No | 18.9% | 7.1% |
| Q19. (Round 2) What should be the maximally length of stay for achieving Textbook Outcome after minimally invasive minor liver resection? | - Less than or equal to 2 days | - | 11.9% |
|  | - Less than or equal to 3 days | - | 28.6% |
|  | - Less than or equal to 4 days | - | 9.5% |
|  | - Less than or equal to 5 days | - | 33.3% |
| Q20. (Round 2) What should be the maximally length of stay for achieving Textbook Outcome after minimally invasive major liver resection? | - Less than or equal to 5 days | - | 16.7% |
|  | - Less than or equal to 6 days | - | 16.7% |
|  | - Less than or equal to 7 days | - | 35.7% |
|  | - Less than or equal to 10 days | - | 21.4% |
| Q21. (Round 2) What should be the maximally length of stay for achieving Textbook Outcome after open minor liver resection? | - Less than or equal to 4 days | - | 28.6% |
|  | - Less than or equal to 5 days | - | 40.5% |
|  | - Less than or equal to 6 days | - | 11.9% |
|  | - Less than or equal to 7 days | - | 4.8% |
| Q22. (Round 2) What should be the maximally length of stay for achieving Textbook Outcome after open major liver resection? | - Less than or equal to 7 days | - | 22% |
|  | - Less than or equal to 8 days | - | 17.1% |
|  | - Less than or equal to 10 days | - | 46.3% |
|  | - Less than or equal to 15 days | - | 7.3% |
| **Domain 6: Oncological Resection Margin** |  |  |  |
| Q23. Which resection margin parameter should be included in the definition of Textbook Outcome in MILS? | - The absence of R1 Resection AND R2 Resection | 59.1% | 92.9% |
|  | - The absence of R2 Resection ONLY | 22.7% | 7.1% |
|  | - Resection margin should not be included in Textbook Outcome | 18.2% | 0 |
| Q24. Which resection margin parameter should be included in the definition of Textbook Outcome in open liver surgery? | - The absence of R1 Resection AND R2 Resection | 61.4% | 92.2% |
|  | - The absence of R2 Resection ONLY | 20.5% | 7.1% |
|  | - Resection margin should not be included in Textbook Outcome | 18.2% | 0% |
| Q25. Should R1 resection margin for colorectal liver metastases be divided into R1 vascular resection and R1 parenchymal resection? | - Yes | - | 64.3% |
|  | - No | - | 31.0% |
|  | - Resection margin should not be included in Textbook Outcome | - | 4.8% |
| Q26. Should oncological resection margin be included for all or selected malignant indications (multiple selections possible)? | - All malignant indications | 88.6% | 90.5% |
|  | - Colorectal liver metastases | 6.8% | 4.8% |
|  | - Hepatocellular Carcinoma | 13.6% | 2.4% |
|  | - Intrahepatic Cholangiocarcinoma | 11.4% | 4.8% |
|  | - Gallbladder carcinoma | 6.8% | 2.4% |
|  | - Non-colorectal liver metastases | 6.8% | 4.8% |
|  | - Oncological resection margin should not be included in Textbook Outcome | 0% | 2.4% |

**Supplemental Table 4.** Domains and questions presented in round 3 with the corresponding agreement rates.

| **Question** | **Answer option** | **Agreement rate** |
| --- | --- | --- |
| **Domain 3: General postoperative complications** |  |  |
| Q1. Do you agree postoperative complications to be included in the definition of Textbook Outcome in liver surgery? | - Yes | 100% |
|  | - No | 0 |
| Q2. If you agree, should we consider 30-or 90-day postoperative complications in the definition of Textbook Outcome in liver surgery? | - 30-day | 26.8% |
|  | - 90-day | 73.2% |
|  | - Complications should not be included in Textbook Outcome | 0% |
| Q3. If you agree, which Clavien-Dindo Grade or higher should be included in the definition of Textbook Outcome in liver surgery? | - The absence of Complication of Clavien-Dindo IV or higher | 2.4% |
|  | - The absence of Complication of Clavien-Dindo III or higher | 63.4% |
|  | - The absence of Complication of Clavien-Dindo II or higher | 34.1% |
|  | - Complications should not be included in Textbook Outcome | 0 |
| Q4. Do you think Readmission should be included in the definition of Textbook Outcome in Liver Surgery? | - Yes | 100% |
|  | - No | 0% |
| Q5. If yes, should we consider Readmission due to all reasons or just surgery related complications? | - All reasons | 48.8% |
|  | - Just surgery related complications of Clavien-Dindo II or higher | 19.5% |
|  | - Just surgery related complications of Clavien-Dindo III or higher | 31.7% |
|  | - Readmission should not be included in Textbook Outcome | 0% |
| Q6. If yes, within what period should we consider readmission as an event? | - 30 days | 39% |
|  | - 90 days | 61% |
|  | - Readmission should not be included in Textbook Outcome | 0% |
| **Domain 4: Liver surgery-related postoperative complications** |  |  |
| Q7. It has been agreed that postoperative bile leakage should be included in the definition of Textbook Outcome in Liver Surgery. However, we do not have an agreement yet on what grade of bile leakage should be considered. Please find attached the grading of bile leakage and please select which grading should be included to reach Textbook Outcome in Liver Surgery. | - The absence of bile leakage of all grades | 39% |
|  | - The absence of bile leakage of grade B & C | 61% |
|  | - The absence of bile leakage of grade C | 0% |
| Q8. It has been agreed that postoperatie liver failure should be included in the definition of Textbook Outcome in Liver Surgery. However, we do not have an agreement yet on what grade of liver failure should be considered. Please find attached the grading of liver failure and please select which grading should be included to reach Textbook Outcome in Liver Surgery. | - The absence of Postoperative liver failure of all grades | 29.3% |
|  | - The absence of Postoperative liver failure of grade B & C | 70.7% |
|  | - The absence of Postoperative liver failure of grade C | 0% |
| **Domain 5: Length of Hospital Stay** |  |  |
| Q9. What should be the maximally length of stay for achieving Textbook Outcome after MINIMALLY INVASIVE MINOR liver resection? | - less than or equal to 3 days | 56.1% |
|  | - less than or equal to 5 days | 43.9% |
| Q10. What should be the maximally length of stay for achieving Textbook Outcome after MINIMALLY INVASIVE MAJOR liver resection? | - less than or equal to 5 days | 73.2% |
|  | - less than or equal to 7 days | 26.8% |
| Q11. What should be the maximally length of stay for achieving Textbook Outcome after OPEN MINOR liver resection? | - less than or equal to 4 days | 24.4% |
|  | - less than or equal to 5 days | 46.3% |
|  | - less than or equal to 6 days | 29.3% |
| Q12. What should be the maximally length of stay for achieving Textbook Outcome after OPEN MAJOR liver resection? | - less than or equal to 8 days | 48.8% |
|  | - less than or equal to 10 days | 51.2% |

**Supplemental Table 5.** Domains and questions presented in round 4 with the corresponding agreement rates.

| **Question** | **Answer option** | **Agreement rate** |
| --- | --- | --- |
| **Domain 3: General postoperative complications** |  |  |
| Q1. It has been agreed that postoperative complications should be included in the definition of Textbook Outcome in Liver Surgery. However, we do not have an agreement yet on the period and what grade of complications should be considered. Should we consider 30-day postoperative complications in the definition of Textbook Outcome in liver surgery? | - Yes | 58.5% |
|  | - No | 41.5% |
| Q2. Should we consider 90-day postoperative complications in the definition of Textbook Outcome in liver surgery? | - Yes | 73.2% |
|  | - No | 26.8% |
| Q3. Should we consider Clavien-Dindo Grade 2 or higher with regards to postoperative complications? | - Yes | 43.9% |
|  | - No | 56.1% |
| Q4. Should we consider Clavien-Dindo Grade 3 or higher with regards to postoperative complications? | - Yes | 80.5% |
|  | - No | 19.5% |
| Q5. Should we consider Clavien-Dindo Grade 4 or higher with regards to postoperative complications? | - Yes | 65.9% |
|  | - No | 34.1% |
| Q6. It has been agreed that readmission should be included in the definition of Textbook Outcome in Liver Surgery. However, we do not have an agreement yet on the period and what reason of readmission should be considered. Should we consider Readmission due to all reasons? | - Yes | 58.5% |
|  | - No | 41.5% |
| Q7. Should we consider Readmission due to surgery related complications Clavien-dindo Grade 2 or higher? | - Yes | 53.7% |
|  | - No | 46.3% |
| Q8. Should we consider Readmission due to surgery related complications Clavien-dindo Grade 3 or higher? | - Yes | 68.3% |
|  | - No | 31.7% |
| Q9. Should we consider Readmission within 30 days? | - Yes | 58.5% |
|  | - No | 41.5% |
| Q10. Should we consider Readmission within 90 days? | - Yes | 63.4% |
|  | - No | 36.6% |
| **Domain 4: Liver surgery related postoperative complications** |  |  |
| Q11. It has been agreed that postoperative bile leakage should be included in the definition of Textbook Outcome in Liver Surgery. However, we do not have an agreement yet on what grade of bile leakage should be considered. Should we consider bile leakage grade B and C? | - Yes | 87.8% |
|  | - No | 12.2% |
| Q12. Should we consider bile leakage of all grades? | - Yes | 31.7% |
|  | - No | 68.3% |
| Q13. It has been agreed that postoperative liver failure should be included in the definition of Textbook Outcome in Liver Surgery. However, we do not have an agreement yet on what grade of liver failure should be considered. Should we consider liver failure grade B and C? | - Yes | 90.2% |
|  | - No | 9.8% |
| Q14. Should we consider liver failure of all grades? | - Yes | 19.5% |
|  | - No | 80.5% |
| **Domain 5: Length of Hospital Stay** |  |  |
| Q15. Length of stay should not be included in the main definition of TOLS. | - Agree | 73.2% |
|  | - Disagree | 26.8% |
| Q16. Length of stay should be included in an extended definition of TOLS (TOLS+). | - Agree | 70.7% |
|  | - Disagree | 29.3% |
